# MetaboVariation 2.0: Multivariate analysis for identifying metabolite variation at the individual level

**DOI:** 10.1371/journal.pone.0343973

**Published:** 2026-05-18

**Authors:** Shubbham Gupta, Isobel Claire Gormley, Lorraine Brennan

**Affiliations:** 1 School of Agriculture and Food Science, University College Dublin, Dublin, Ireland; 2 School of Mathematics and Statistics, University College Dublin, Ireland; Central University of Punjab, INDIA

## Abstract

Variations in individuals’ metabolic profiles are the result of their genetic makeup and environmental and lifestyle factors. To address the challenge of identifying these intra-individual variations at the individual level, we introduce “MetaboVariation 2.0”, a multivariate Bayesian generalised linear model designed to flag individuals with intra-individual variations in metabolite levels across repeated measurements. MetaboVariation 2.0 builds upon the previous univariate MetaboVariation approach by incorporating dependencies between metabolites, offering a more comprehensive assessment of individual metabolic variations. While simultaneously considering all metabolites, MetaboVariation 2.0 flags an individual when their observed metabolite levels deviate from their individual-level posterior predictive interval at a time point. A series of simulation studies were conducted to evaluate the performance of MetaboVariation 2.0. In addition it was applied to a metabolomics data set. The efficacy of this approach was validated through a series of simulation studies. These simulations demonstrated that the multivariate model outperformed its predecessor, particularly in scenarios where the dependencies between the metabolites were positive. The model showed lower mean absolute differences between correlation matrices of metabolite levels from replicate datasets and the original simulated data, indicating improved accuracy in capturing the metabolic dependencies. In addition, analysis of plasma metabolite levels from 164 individuals with 20 metabolites measured across four time points was performed to detect individuals with intra-individual variations. MetaboVariation 2.0 revealed intra-individual variations in 15.2% of the individuals, with 20% or more of their metabolites showing variations beyond their 97.5% posterior predictive intervals in at least one time point. In conclusion, MetaboVariation 2.0 accounts for the inherent dependencies between different metabolites, offering a full view of an individual’s metabolic profile which is an important advancement for assessment of individual-level metabolite variation. A software implementation of this approach is freely available through the “MetaboVariation” R package, promoting its accessibility and use in broader metabolomics research.

## Introduction

Metabolomics measures metabolites present in biological systems, providing valuable insights into the biochemical functions and physiological state of living organisms. By identifying and quantifying small molecule metabolites, metabolomics provides an indirect representation of the complex network of biochemical pathways and their interactions within a system [1–5]. Furthermore, metabolomics studies highlight the importance of correlations between metabolites [6,7]. In recent years, personalised medicine has emerged, with metabolomics playing a role in identifying profiles that predict responses to clinical treatment. [8–10]. A previous study [11] analysed urine samples from 20 children three times a day for six days, quantifying 44 metabolites, and found significant inter- and intra-individual variability in metabolic profiles, with a median intraclass correlation coefficient of 0.40. Examination of urine profiles revealed that the genetic and environmental background of a person influences the metabolic profile [12]. Further research [13] has demonstrated that while plasma metabolite levels generally exhibit high reliability and stability over time, there are also some intra-individual variations, particularly in certain metabolites. Examining these repeated samples and changes over time is likely to be useful in epidemiological studies [13].

Recent progress has observed a paradigm shift towards personalised diagnostic and therapeutic strategies, facilitated by the implementation of personalised reference intervals (prRIs) [14,15]. These account for inherent individual metabolite variations, diverging from conventional universal reference ranges. By understanding metabolite variations from previously analysed laboratory results, and data on analytical and within-subject biological variations, reliable prRIs can be constructed with three or more previously analysed test results, thus improving patient evaluation and therapeutic monitoring. Recent research [16,17] has introduced the concept of individual reference intervals (IRI), which provide a personalised reference range based on the biological characteristics of an individual. Unlike prRIs, which are tailored using previously analysed results of individuals, IRIs are constructed using quantile models that consider both inter- and intra-individual variability over time. These approaches collectively emphasise the importance of personalised metrics in capturing unique metabolic profiles, thus supporting the development of personalised healthcare strategies. While prRIs and IRIs are effective at creating reference intervals, they focus on metabolites independently and do not account for the interdependencies between the metabolite levels.

The objective of this study is to develop an approach for analysing intra-individual variation in metabolite data that accounts for the inherent dependencies between metabolite levels. Building on previous work with the univariate MetaboVariation approach [18], a multivariate Bayesian generalised linear model (BGLM) is used. This model flags individuals by simultaneously considering all metabolites and their dependencies, rather than focussing on a single metabolite. Individuals are flagged if their observed metabolite levels deviate from their individual posterior predictive intervals at a specific time point. The novelty of this multivariate approach lies in its ability to model multivariate metabolite data at the individual level, accounting for dependencies between metabolites when identifying intra-individual variation in metabolite levels.

The remainder of this paper discusses the multivariate MetaboVariation 2.0 approach (Section Materials and methods), evaluates its performance using simulated and real-world data (Section Results), and discusses its use and potential enhancements (Section Discussion). All reported results were generated using the “MetaboVariation” R package, which is available at github.com/shubbham28/MetaboVariation for wider distribution.

## Materials and methods

### Metabolomics data

The current study uses metabolomics data taken from plasma samples in the A-Diet Confirm study [19,20], which received ethical approval from the University College Dublin Sciences Human Research Ethics Committee (LS-16–91-Gibbons-Brennan). All participants gave written informed consent and were recruited between 14th January 2017 and 8th October 2018. The A-Diet Confirm study aimed to investigate the regular dietary intake of participants over a period of four months. This involved the monthly collection of biological samples and dietary information. The current study specifically used amino acid data from a previously published data set [19]. Data were generated using the Biocrates AbsoluteIDQ p180 kit on a Sciex QTRAP 6500 + mass spectrometer with a UHPLC column. Amino acids were quantified with isotopically labelled internal standards and seven-point calibration curves in the AB Sciex Analyst version 1.7.2 software. Fasting plasma samples were collected monthly for four consecutive months, reflecting the regular lifestyle habits of the participants. From the primary data set, M=20 metabolites were selected and N=164 participants provided a maximum of T=4 repeated measurements. In addition to metabolite data, L=3 covariates (sex, age, and BMI) were recorded for each individual. The mean BMI was 24.1±3.07 kg/m^2^, and the mean age was 35±12.6 years, with 53 male and 111 female participants.

The second application is from published data from a disease model of Duchenne muscular dystrophy which included longitudinal data from wild type and disease mice [21]. A total of 106 metabolites (M=106) were available in the dataset over three timepoints (T=3 (6, 12, 18 weeks). Three timepoints were used as data was missing from 4 animals at weeks 18 and 24.) Data were available for a total of N=10 animals.

## The multivariate metabovariation model

Expanding on the previous univariate MetaboVariation approach [18], where metabolites were independently analysed, here in MetaboVariation 2.0, a multivariate Bayesian generalised linear model (BGLM) is used that accounts for the dependencies between the metabolites. In the MetaboVariation 2.0 model, metabolites are assumed to have a dense covariance matrix rather than a diagonal covariance matrix, as is the case in the independent setting. By incorporating a dense covariance matrix into the statistical modelling, the inherent dependencies between metabolites are taken into account, facilitating a more comprehensive view of repeated measurements of metabolites within individuals.

The observed data contain the levels of *M* metabolites of *N* individuals at *T* time points, organised into a matrix Y of dimension (N×T)×M. The multivariate MetaboVariation 2.0 model is


Y=Xβ+S+ϵ (1)

where the matrix X contains a column of *1*s for the intercept and the *L* covariates, the (L+1)×M matrix β contains the regression coefficients and the matrix S captures the random effects of all individuals at each time point and each metabolite. Here, S=Zu where Z is the (N×T)×(N×M) binary design matrix and u contains the random effects for the metabolites. The term ϵ represents the random error associated with each measurement.

In 1 the fixed effects (β), random effects (u), and residuals (ϵ) are assumed to follow a multivariate normal distribution, i.e.,


$$\begin{array}{c} (\begin{array}{c} \beta \\ u\\ \epsilon \end{array})\sim 𝒩\left((\begin{array}{c} \beta_{0}\\ 0\\ 0 \end{array}),(\begin{array}{ccc} \text{B} & 0 & 0\\ 0 & \text{G} & 0\\ 0 & 0 & \text{R} \end{array})\right). \end{array}$$ (2)

The fixed effects (β) follow a multivariate normal prior distribution with mean $\beta_{0}$ and prior covariance matrix B . Here, the values of the hyperparameters $\beta_{0}$ and B  are informed by the result of fitting a univariate BGLM to each metabolite independently: the mean $\beta_{0}$ is set to the posterior mean of the regression coefficients obtained from these univariate BGLMs, and the diagonal matrix B  is set to the variances of the posterior distributions of the regression coefficients. For the random effects (u) and residuals (ϵ), prior means of *0* along with dense covariance matrices G  and R , respectively, are assumed; G  and R  have inverse Wishart prior distributions [22]. These prior distributions are assumed because of their flexibility and conjugate properties. The inverse Wishart distribution is characterised by two hyperparameters: the scale matrices $\Sigma^{2}$ and $\Sigma_{\epsilon }^{2}$ for random effects G  and residuals R , respectively, and the degrees of freedom *ν* for both G  and R . Here, the diagonal elements of the scale matrices $\Sigma^{2}$ and $\Sigma_{\epsilon }^{2}$ are set to the variances of the posterior distributions of the random effects and residuals, respectively, as obtained from fitting a univariate BGLM to each metabolite independently. The off-diagonal entries of the scale matrices are presumed to have an absolute value of 0.1, with the signs of these off-diagonal terms fixed to match the signs from the correlation matrix of the observed metabolite data Y. As in [22], the degrees of freedom in the priors for G  and R  are assumed to be 150% of the number of metabolites *M*. The chosen hyperparameters were selected so that the prior faithfully reflects dependencies in the data while being relatively non-informative. A prior sensitivity analysis was performed (see Appendix 1 in S1 File) to examine the robustness of the inference under different hyperparameter settings. The analysis revealed negligible difference in inference under the hyperparameter settings considered.

After fitting the multivariate BGLM using the MCMCglmm R package [22] via Metropolis-Hastings algorithm [23], a posterior predictive distribution is constructed for each individual i=1,…,N for each time point t=1,…,T and for each metabolite m=1,…,M. The validity of these posterior predictive distributions is ensured by monitoring the convergence of the Metropolis-Hastings algorithm; here, the potential scale reduction factor (PSRF) was used [24] to assess convergence. After constructing each individual’s posterior predictive distribution, a highest posterior density (HPD) prediction interval is determined for each metabolite m=1,…,M and for each time point t=1,…,T. If the observed level of metabolite *m* for individual *i* at time point *t* does not reside within the relevant HPD interval of the individual, the individual is flagged, indicating intra-individual variation in the level of metabolite *m* at time point *t* for individual *i*. Different widths of HPD interval could be considered; here 95%, 97.5%, and 99% are used. In addition, radar plots are used to visualise intra-individual variation in the metabolic profile of an individual across all time points and metabolites, by displaying the individual’s observed metabolite levels with the observed metabolite levels of the study cohort, alongside the individual’s posterior predictive intervals for each metabolite and time point.

## Simulation study

A simulation study is conducted to assess how well the MetaboVariation 2.0’s multivariate model performs under various metabolite correlation structures and to compare it to its predecessor, MetaboVariation’s independent model. Parameters estimated from the A-Diet Confirm study data are used to simulate the data to ensure realistic simulation conditions.

Three scenarios are considered in the simulation study, each involving T=4 repeated measurements of M=5 metabolites for a cohort of N=150 individuals, each with L=3 covariates. Each simulated data set Y is generated based on (1), where the covariate matrix X is comprised of two covariates simulated from Gaussian distributions (reflecting age and BMI), and a third covariate simulated from a Bernoulli distribution (reflecting sex). To explore performance under different metabolite correlation structures, different settings of β and the covariance matrices G  and R , of the random effects u and residuals ϵ respectively, are considered in each of the three scenarios.

Scenario 1: This scenario evaluates the performance of MetaboVariation 2.0 when dealing with data that exhibit positive dependencies between metabolites. The multivariate BGLM was fitted to five metabolites from the A-Diet Confirm study that exhibited positive correlations ranging from 0.1 to 0.9, with half of the correlations lying between 0.5 and 0.9. Resulting posterior means of β, **G** and **R** were then used to simulate a data set Y, based on (1) and on the zero-centred multivariate normal distributions of u and ϵ detailed in (2).

Scenario 2: This scenario assesses MetaboVariation 2.0’s performance when there are no dependencies between metabolites. The simulated data are generated as in scenario 1, but where the the off-diagonal elements of G  and R  are set to zero.

Scenario 3: This scenario examines MetaboVariation 2.0’s performance when the dependencies between the metabolites in the simulated data mirror those present in the A-Diet Confirm study data. The multivariate BGLM was fitted to five metabolites from the A-Diet Confirm study that exhibited a mixture of negative and positive correlations ranging from −0.6 to 0.6, with more than half of the correlations falling between −0.3 and 0.3. Again, resulting posterior means of β, **G** and **R** were used to simulate a data set Y, based on (1) and (2). This scenario evaluates MetaboVariation 2.0’s performance in the presence of a mixture of negative and positive dependencies, reflecting the diverse correlation structure observed in the A-Diet Confirm study data.

For each scenario, 25 simulated data sets are generated, and both the independent and multivariate models are fitted to them. Subsequently, 100 replicate data sets are derived from the posterior predictive distributions under both models. To evaluate the performance of the models, the mean absolute differences (MAD) were calculated between the correlation matrices of metabolite levels from the replicate and original simulated data sets at all time points. This evaluation provides an assessment of MetaboVariation 2.0’s performance under different metabolite dependency patterns.

## Results

### Simulation study results

**Fig 1:**
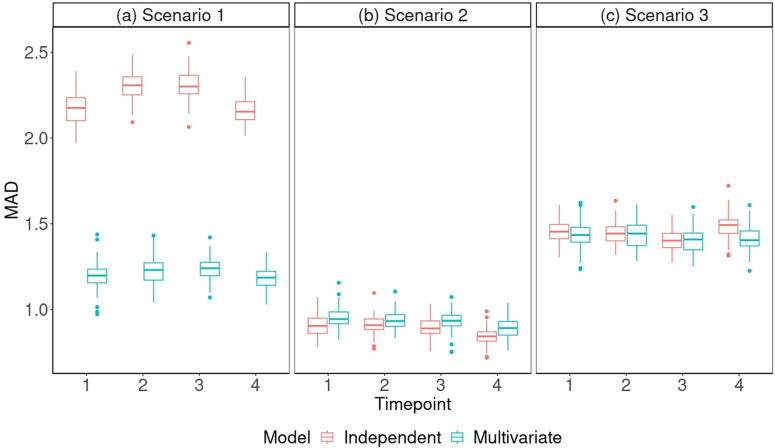
The MAD between correlation matrices of replicate data and simulated data in the simulation scenarios. Boxplots illustrating the MAD between correlation matrices of 100 replicate posterior predictive data sets and the simulated data set at each time point. Results are shown for three simulation scenarios with different levels of dependency between five metabolites: (a) scenario 1 (positive dependencies), (b) scenario 2 (no dependencies), and (c) scenario 3 (mixture of negative and positive dependencies).

To evaluate the performance of the multivariate and independent models, the box plots in Fig 1 illustrate the MAD between the correlation matrices of the simulated data and the replicate posterior predictive data sets at each time point in three simulation scenarios.

In scenario 1, where the metabolites exhibit positive correlations, Fig 1 (a) shows that the multivariate model has lower MAD values compared to the independent model. This indicates that the multivariate model is better at capturing the inherent dependencies present in the metabolite data particularly when the metabolites are positivelt correlated. Importantly, Fig 1 (a) also highlights that fitting an independent model in the presence of such dependencies will result in incorrect inference.

The similar MAD ranges for the multivariate and independent models in scenario 2 suggest that MetaboVariation 2.0 performs effectively in situations where there are no dependencies between the metabolites. For scenario 3, which replicates the positive and negative dependencies observed between metabolites in the A-Diet Confirm study, the MAD values shown in Fig 1(c) suggest that the multivariate model performs effectively and similarly to the independent model. However, the median MAD at each time point in the multivariate model is equal to or lower than that in the independent model. The multivariate model demonstrates its ability to account for the observed variety of metabolite correlation patterns through its low MAD values.

## Metabolomics data

**Fig 2:**
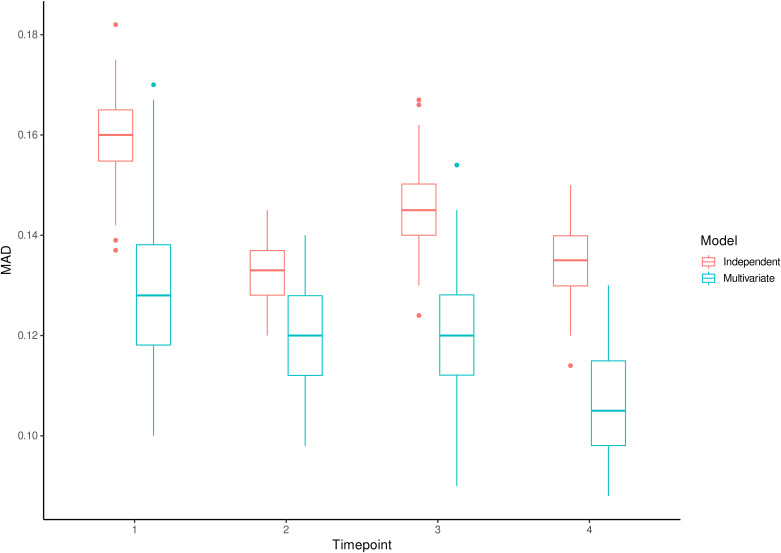
The MAD values between correlation matrices of replicate data from the fitted model and plasma metabolites data. Boxplots of the MAD values between correlation matrices of 100 posterior predictive replicate data sets from the multivariate and independent models and observed plasma metabolites data of the A-Diet Confirm study at different time points.

Comparison of the multivariate and independent models fitted to the plasma metabolite data from the A-Diet Confirm study revealed lower MAD values for the multivariate model (Fig 2). Consequently, the multivariate model is used to flag individuals with intra-individual variation in metabolite levels.

MetaboVariation 2.0 was used to flag individuals with intra-individual variation by considering three HPD widths: 95%, 97.5%, and 99%. Intuitively, there is a decrease in the number of individuals flagged as the interval widens. The proportion of individuals flagged with intra-individual variation in at least four metabolites, in a time point, is 28.1% when 95% HPD intervals are considered. This percentage decreased to 15.2% with 97.5% HPD intervals and to 7.3% with 99% HPD intervals. A similar decreasing trend was observed when considering individuals with intra-individual variations in at least five metabolites. MetaboVariation 2.0 flagged 19.5% of individuals with intra-individual variations in at least five metabolites with 95% HPD intervals, which decreased to 9.7% and 4.2% with 97.5% and 99% HPD intervals, respectively.

**Fig 3:**
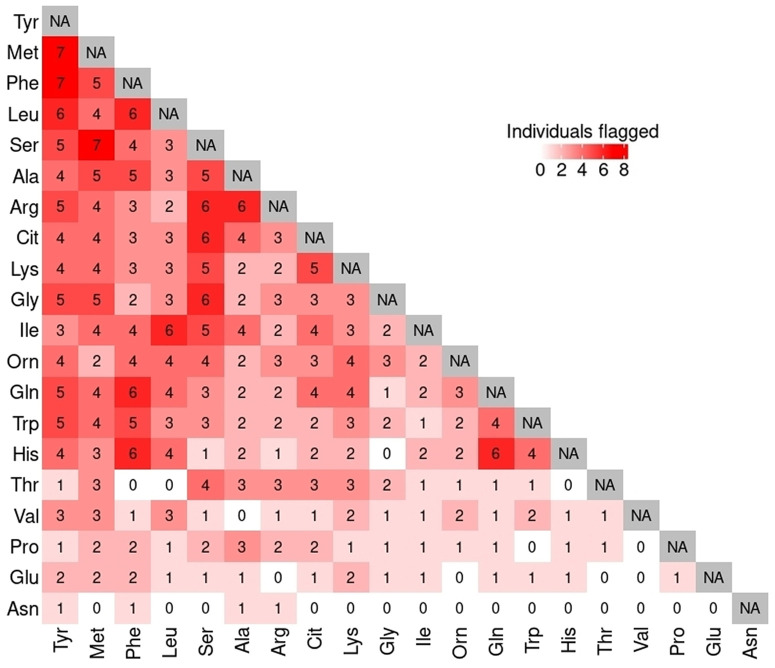
Number of flagged individuals for metabolite pairs. A heat map illustrating the number of flagged individuals in which the pair of metabolites (in the corresponding row and column) contributes to flagging the individuals within a time point.

The heat map in Fig 3 illustrates the number of flagged individuals for whom pairs of metabolites contribute to intra-individual variation within a time point, with darker shades of red indicating a higher number of flagged individuals. The heatmap in Fig 3 refers to the subset of individuals that are flagged for at least five metabolites within a time point. Of the 21 individuals flagged for at least five metabolites, 7 individuals were flagged in multiple metabolite pairs, including Met and Tyr, Phe and Tyr, and Ser and Met. Six individuals were flagged for having intra-individual variation in 10 different pairs of metabolites, while five individuals shared 14 pairs of metabolites in which those individuals were flagged. This highlights the importance of a comprehensive multivariate analysis of the total metabolic profile to understand the dependencies between metabolites when flagging individuals with intra-individual variations.

**Fig 4:**
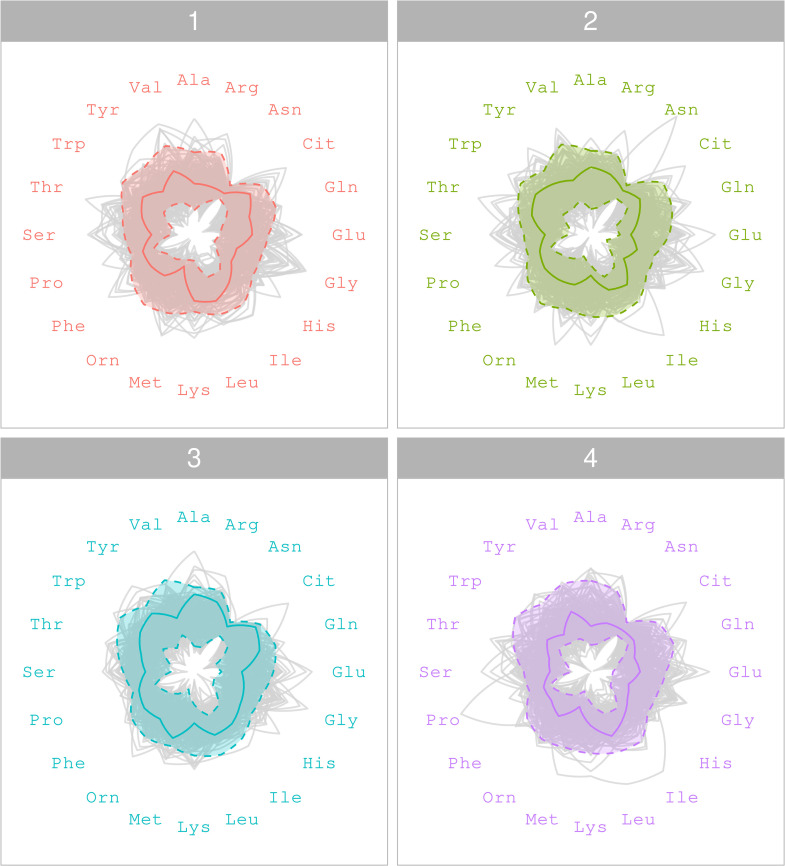
Radar plot of plasma metabolite levels for a non-flagged individual. A radar plot showing the plasma metabolite levels of a typical, non-flagged individual from the A-Diet Confirm study at T=4 time points. The shaded regions denote the 97.5% HPD intervals of the individual’s posterior predictive distribution, while the light grey lines illustrate the metabolite levels of the entire study cohort.

**Fig 5:**
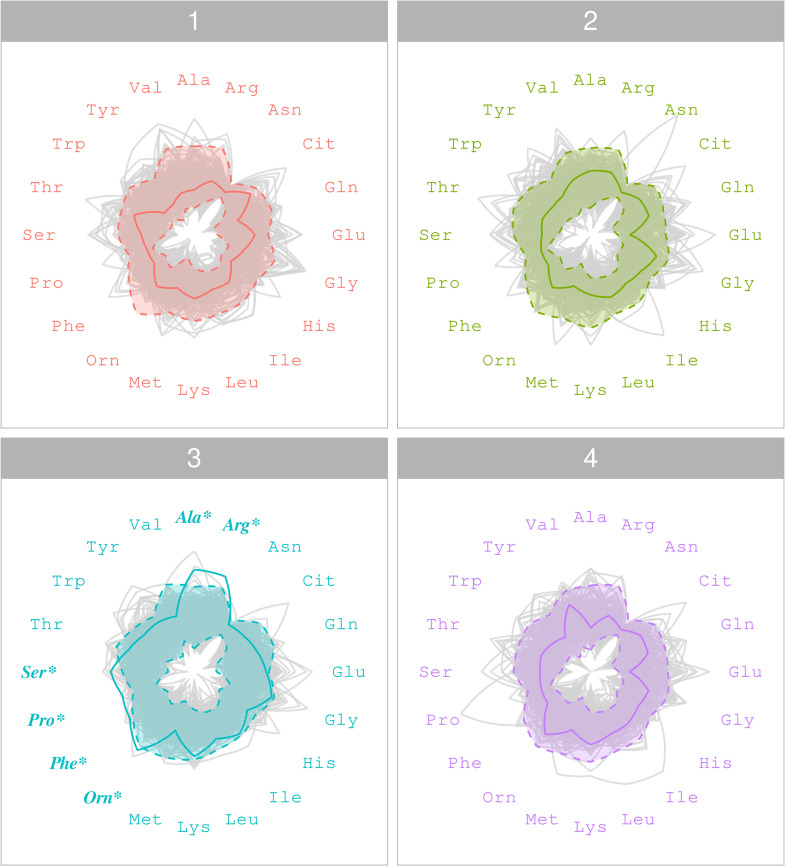
Radar plot of plasma metabolite levels with intra-individual variation. A radar plot illustrating the plasma metabolite levels of an individual from the A-Diet Confirm study at T=4 time points. The shaded regions denote the 97.5% HPD intervals of the individual’s posterior predictive distribution and light grey lines signify the metabolite levels of the entire study cohort. Intra-individual variation is visible in six metabolites (indicated by bold font and *) at the third time point as the individual‘s observed metabolite levels exceed their 97.5% HPD intervals.

Typical variations in metabolite levels across time points in a non-flagged individual are shown in the radar plot in Fig 4. The radar plot shown in Fig 5 illustrates the metabolite levels of a flagged individual; notably, at the third time point, intra-individual variation is observed in six metabolites (Ala, Arg, Orn, Phe, Pro and Ser). Although metabolite levels at time points one, two, and four fall within the 97.5% HPD intervals, some metabolite levels at the third time point show noticeable variation.

When applied to the data from the A-Diet Confirm study, MetaboVariation 2.0, running on a Dell Latitude 5511 laptop, took approximately twenty minutes across four chains of the Metropolis-Hastings algorithm with 5000 iterations each, including a 2000 iteration burn-in period, with a thinning rate of 3. The chains were deemed to be converged with observed PSRF values of 1.0005 (standard deviation 0.002). Processing time may vary according to the specifications of the system, the number of chains in the Metropolis-Hastings algorithm, the iterations, and the size of the data set.

Application to the metabolomics data from the disease model of Duchenne muscular dystrophy highlighted 5 metabolites with variation at 80% HPD intervals. These included R-butyryl carnitine, anserine, nicotinamide, o-Phosphorylethanolamine and uracil. These metabolites map to metabolic pathways that overlap with the identified pathways included in the original paper highlighting the potential for MetaboVariation 2.0 in applications of disease models [21].

## Discussion

Personalised healthcare, which tailors recommendations to individual characteristics, has attracted significant attention [25–29]. In recent years, emphasis has been placed on omics technologies such as metabolomics, metagenomics, proteomics, and transcriptomics, which have emerged as key tools to achieve personalised health goals [30]. Recent research has highlighted the importance of examining variations within individuals and, therefore, the importance of personalised metabolic profiles in predicting health results and response to external factors [11,31]. In response to the challenge of analysing intra-individual variations in metabolite levels, an improved approach to MetaboVariation, which uses an independent model for each metabolite, is presented. The improved approach, MetaboVariation 2.0, analyses repeated measurements of metabolite levels for individuals more appropriately by taking the dependencies between the metabolites into account when flagging individuals showing variation. In addition, an R package named “MetaboVariation” [32] was created to facilitate widespread application of the method.

Existing research often depends on metrics like the coefficient of variation (CV) and intraclass correlation coefficients (ICCs) to analyse inter- or intra-individual variations. Previous studies [13,33] have effectively used these methods to assess the reliability of biomarkers in longitudinal metabolite data. Although these methods offer valuable information, they typically analyse each biomarker independently, potentially missing the complex interdependencies that exist between multiple biomarkers. Previously, a univariate MetaboVariation approach [18], flagged intra-individual variation that was missed by an ICC approach, while complementing conclusions drawn from ICCs in some cases. MetaboVariation 2.0 provides a more nuanced approach by simultaneously accounting for interdependencies between multiple biomarkers and thus analysing how multiple metabolites vary together within individuals over time at the individual level. Other approaches, such as functional data analyses [34] and clustering based methods [35], could also prove fruitful research avenues for assessing metabolite variation at the individual level.

Examining plasma data from the A-Diet confirm study [19], MetaboVariation 2.0 flagged individuals with intra-individual variation in metabolite levels. To allow flexibility in the application we demonstrated that the number of individuals flagged will vary according to the HPD level chosen: this selection will depend on the application. If a user is interested in individuals with extreme intra-individual variation then a 99% HPD interval is useful and in the A-Diet application flagged 7.3% of the individuals. In addition, examining the frequency of pairs of metabolites being flagged highlights the importance of using the full metabolic profile to flag individuals instead of a single metabolite, as used in the univariate predecessor. The majority of metabolites were involved in the flagging of the individuals, with only one metabolite (Asn, see Fig. 3) having a minimal role. Radar plots are used to visualise intra-individual variation in the metabolic profile of an individual across all time points by displaying the individual’s observed metabolite levels along with those of the study cohort. The use of radar plots in the A-Diet application is insightful as they allow for simultaneous visualisation of all metabolites for an individual alongside those of the entire cohort and clearly communicate the HPD intervals; this assists the user in visualising the flagged intra-individual variation. However, the need for separate radar plots for each time point, along with their lack of scalability in settings with large numbers of metabolites could limit their utility in other settings. Parallel coordinates plots [36] may provide a useful alternative in such settings. By accounting for the dependencies between metabolites, MetaboVariation 2.0 enhances the analysis of intra-individual variations in metabolites, providing better fitting posterior predictive intervals compared to the earlier version. Our context for the application of MetaboVariation 2.0 was a healthy population group with a focus on intra-individual variation so that individuals with variation over time could be flagged. This could identify individuals with early alterations in metabolism that could be flagged for early lifestyle intervention to slow the progression of disease. The application to the animal model data highlighted the potential of the approach in the disease setting.

Although this study addresses the limitations related to ignoring metabolite dependencies, some limitations remain. For example, although the multivariate approach was applied to a set of twenty metabolites, as the number of metabolites and observations increases, the model will encounter challenges related to the extended computational time. Settings with larger datasets are likely to be practically challenging for MetaboVariation 2.0, both in terms of run times and interpretation of the resulting radar plots and heat maps. However, more computationally efficient inferential methods, such as variational or approximate Bayesian inference, sparse modelling approaches or variable (i.e., metabolite) selection methods, may help to address this challenge. Moreover, the current approach assumes that the metabolite levels are Gaussian distributed, which may not be an optimal model. To improve model fit, other distributions such as the truncated Gaussian [37] or heavier-tailed distributions such as the (skew) t-distribution [38] could be employed in the model specification, better capturing the truncated or skew distributions of metabolites respectively. Further, a zero-inflated version of the MetaboVariation 2.0 model, similar to [39], could be developed to account for zero-inflated metabolite data. Fitting such modified MetaboVariation 2.0 models may be more computationally expensive than fitting the Gaussian specification, but in return for an improved model fit.

Most biomarker research to date has focused on improving disease diagnosis by comparing with disease individuals with control groups. However, these methods have limited efficacy for the early detection of biomarkers related to disease or metabolic problems. Future research will focus on flagging individuals with intra-individual variation and then investigating whether these individuals may be at risk of developing certain diseases in the future or not. If successful the application could be incorporated into personalised monitoring and could form part of early disease screening. If deviations in metabolism could be identified early, lifestyle interventions could be implemented in a timely fashion. Future work will develop the evidence base for such approaches.

## Conclusion

This study improves the previous MetaboVariation approach by accounting for dependencies between metabolites. This allows for a more faithful evaluation of intra-individual variations in metabolite levels within individuals. The capability of MetaboVariation 2.0 to capture these dependencies is confirmed using both simulated and real-world data. MetaboVariation 2.0 is used to flag individuals with intra-individual variation by utilising their personalised posterior predictive distributions. These improvements in metabolomics analysis at the individual level represent a significant step forward in personalised healthcare. The purpose of making MetaboVariation 2.0 available as open-source software is to promote its use and ensure easy access for a wide range of users.

## Supporting information

S1 File Supporting information 1 for ‘MetaboVariation 2.0: multivariate analysis to identify metabolite variation at the individual level‘ data by Gupta et al. Supporting information includes prior sensitivity analysis of hyperparameters for the multivariate model.
(PDF)
