# Supplementary material for: MetaboVariation 2.0: Multivariate analysis for identifying metabolite variation at the individual level
Source: PLoS One. 2026 May 18;21(5):e0343973. doi: 10.1371/journal.pone.0343973 (PMC13183246; doi:10.1371/journal.pone.0343973)
Supplement: S1 File — Supporting information includes prior sensitivity analysis of hyperparameters for the multivariate model. (PDF) [file pone.0343973.s001.pdf]

Supporting Information 1 for 'MetaboVariation 2.0: multivariate analysis for identifying metabolite variation at the individual level' data by Gupta et al.

Appendix S1

To assess the robustness of the MetaboVariation 2.0 framework, a prior sensitivity analysis is performed. The goal is to examine the robustness of the inference of the model under different specifications of hyperparameters in the inverse Wishart distributions, specifically in terms of the ability to replicate the correlation structure of the metabolite data.

Eighteen scenarios with different hyperparameter settings were considered. Specifically, three values of ν were used: $\{1.1 \times M, 1.5 \times M, 2 \times M\}$, where M represents the number of metabolites in the data. The diagonal elements of the scale matrices $\mathbf{\Sigma}^2$ and $\mathbf{\Sigma}_\epsilon^2$ were set to be from the variances of the posterior distributions of random effects and residuals, respectively, obtained from fitting a univariate BGLM to each metabolite independently. For the off-diagonal values of the scale matrices, values in the set $\{0.01, 0.1, 0.5\}$ were considered. Two further settings for the off-diagonal terms were also considered: one in which all off-diagonal values were positive and another in which the signs of the scale matrix matched the signs from the correlation matrix of the metabolite data. Table 1 summarises the hyperparameter settings considered.

Hyperparameter settings were evaluated by fitting the MetaboVariation 2.0 approach to data from the A-Diet Confirm study. A total of 500 replicate data sets were generated from posterior predictive distributions under the fitted model. The mean absolute difference (MAD) between the correlation matrix of the observed data and the replicate data sets was calculated for each hyperparameter setting.

Table 1. Different hyperparameter settings considered.

Scenario	ν	Off-diagonal values
1	1.1×20	0.01
2	1.1×20	0.1
3	1.1×20	0.5
4	1.5×20	0.01
5	1.5×20	0.1
6	1.5×20	0.5
7	2×20	0.01
8	2×20	0.1
9	2×20	0.5
10	1.1×20	0.01, sign fixed
11	1.1×20	0.1, sign fixed
12	1.1×20	0.5, sign fixed
13	1.5×20	0.01, sign fixed
14	1.5×20	0.1, sign fixed
15	1.5×20	0.5, sign fixed
16	2×20	0.01, sign fixed
17	2×20	0.1, sign fixed
18	2×20	0.5, sign fixed

The MAD values across different hyperparameter settings and time points, as visualised in Fig. 1, indicate negligible differences in the inference. This suggests that the MetaboVariation 2.0 approach is robust to varying the hyperparameters. Scenario 14 with $\nu = 1.5 \times 20$ and off-diagonal values set to 0.1 with the sign fixed as the signs in the observed correlation matrix was selected for use in the main study due to its good performance, on average, across all time points and all scenarios considered.

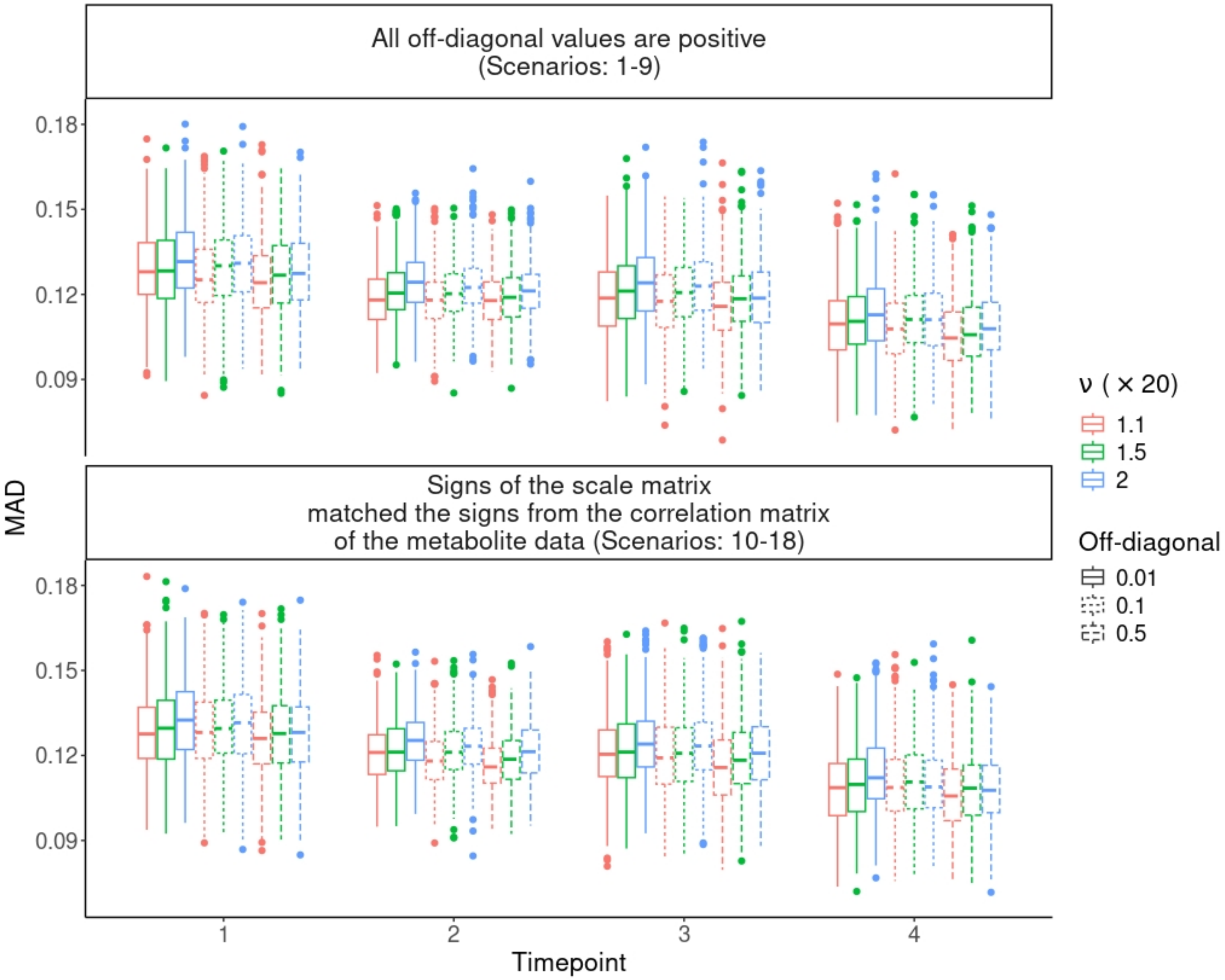

Fig 1. Boxplots illustrating the MAD between the correlation matrices of the observed data from the A-Diet Confirm study and 500 replicate data sets generated from the fitted model's posterior predictive distributions under each hyperparameter setting.
